# Liddle Syndrome: Review of the Literature and Description of a New Case

**DOI:** 10.3390/ijms19030812

**Published:** 2018-03-11

**Authors:** Martina Tetti, Silvia Monticone, Jacopo Burrello, Patrizia Matarazzo, Franco Veglio, Barbara Pasini, Xavier Jeunemaitre, Paolo Mulatero

**Affiliations:** 1 Division of Internal Medicine and Hypertension Unit, Department of Medical Sciences, University of Torino, Via Genova 3, 10126 Torino, Italy; tetti.martina@gmail.com (M.T.); silvia.monticone@unito.it (S.M.); jacopo.burrello@gmail.com (J.B.); franco.veglio@unito.it (F.V.); 2 Department of Pediatric Endocrinology, Regina Margherita Children Hospital, Città della Salute e della Scienza, 10126 Torino, Italy; pmatarazzo@cittadellasalute.to.it; 3 Unit of Medical Genetics, Department of Medical Sciences, University of Torino, Via Genova 3, 10126 Torino, Italy; barbara.pasini@unito.it; 4 INSERM U970, Paris Cardiovascular Research Center, 75015 Paris, France; xavier.jeunemaitre@aphp.fr; 5 Faculty of Medicine, University Paris Descartes, Paris Sorbonne Cité, 75006 Paris, France; 6 AP-HP, Department of Genetics, Hôpital Européen Georges Pompidou, 75015 Paris, France

**Keywords:** hypertension, hypokalemia, low renin hypertension, monogenic hypertension, Liddle syndrome, *SCNN1A*, *SCNN1B*, *SCNN1G*

## Abstract

Liddle syndrome is an inherited form of low-renin hypertension, transmitted with an autosomal dominant pattern. The molecular basis of Liddle syndrome resides in germline mutations of the *SCNN1A*, *SCNN1B* and *SCNN1G* genes, encoding the α, β, and γ-subunits of the epithelial Na^+^ channel (ENaC), respectively. To date, 31 different causative mutations have been reported in 72 families from four continents. The majority of the substitutions cause an increased expression of the channel at the distal nephron apical membrane, with subsequent enhanced renal sodium reabsorption. The most common clinical presentation of the disease is early onset hypertension, hypokalemia, metabolic alkalosis, suppressed plasma renin activity and low plasma aldosterone. Consequently, treatment of Liddle syndrome is based on the administration of ENaC blockers, amiloride and triamterene. Herein, we discuss the genetic basis, clinical presentation, diagnosis and treatment of Liddle syndrome. Finally, we report a new case in an Italian family, caused by a *SCNN1B* p.Pro618Leu substitution.

## 1. Introduction

Arterial hypertension, affecting about one billion people worldwide, is the most prevalent modifiable risk factor for cardiovascular diseases and related disability [1]. Essential hypertension is a multifactorial condition, resulting from a complex interaction between lifestyle and genetic factors. A positive family history increases the overall risk of developing high blood pressure and genetic factors account for 30–50% of the individual risk [2]. A minority of the hypertensive patients are affected by an inherited disease, resulting from single gene germline mutations affecting mineralocorticoid, glucocorticoid or sympathetic pathways [2,3]. Among these diseases, Liddle syndrome (LS) is caused by point mutations of the epithelial sodium channel (ENaC), that cause renal aldosterone-independent sodium reabsorption. The aim of this review is to provide an update on the current knowledge of LS, including the genetic and pathophysiological basis, the clinical features, the diagnostic and medical management and a new case is reported.

## 2. Liddle Syndrome

### 2.1. Historical Description

The first family affected by a new clinical syndrome that mimicked primary aldosteronism was reported by Liddle et al. [4,5] in 1963 (pseudoaldosteronism, subsequently named Liddle syndrome, OMIM #177200). The index case was a 16-year-old Caucasian girl, who presented with low renin resistant hypertension (180/110 mmHg), severe hypokalemia (2.8 mmol/L) and metabolic alkalosis. These features could resemble those of primary aldosteronism, but this disease was ruled out on the basis of suppressed plasma aldosterone. At the time, Liddle et al. conducted many clinical and biochemical analyses in order to further characterize this peculiar disorder. Under the condition of low sodium intake, aldosterone secretion did not increase and urinary sodium excretion rate decreased, but not to the level that would have been expected in a normal subject. Instead, the urinary sodium levels fell maximally after the administration of exogenous aldosterone. These pieces of evidence suggested an inadequate level of plasma aldosterone rather than an intrinsic renal defect in sodium reabsorption as a cause of the incapability to maximally retain sodium. Compared with patients affected by Addison’s disease, subjects with LS presented a lower urinary Na^+^ excretion, indicating a greater renal reabsorption due to a mechanism different from mineralocorticoid activity. Urinary mineralocorticoid and glucocorticoid metabolites resulted within the physiologic range. Notably, spironolactone administration neither modified urinary electrolytes excretion nor corrected hypokalemia, hence an intrinsic renal defect was hypothesized. Instead, the index case responded to the administration of triamterene, an inhibitor of the epithelial Na^+^ channel, that induced an increase in urinary sodium and a reduction in urinary potassium excretion [4,5]. Moreover, the association of triamterene (100 mg every 8 h) and a low sodium diet normalized blood pressure (diastolic blood pressure dropped to 80 mmHg) and hypokalemia (serum K^+^ rose to 5 mmol/L) [4,6]. Considering the biochemical profile and the response to triamterene, Liddle et al. hypothesized that the distal nephron could be the site of sodium retention. The index case developed chronic renal failure due to hypertensive nephrosclerosis and she underwent renal transplantation in 1989. This intervention corrected the disorder, normalizing blood pressure (140/79 mmHg) and the kalemia (4.2 mmol/L). After the transplantation, a regimen of low salt intake resulted in a normal increase in plasma renin activity and in plasma aldosterone concentration [6]. In 1994, Botero-Velez et al. described the extended pedigree of the family reported by Liddle et al., thus, demonstrating the autosomal dominant inheritance of the disorder [6]. Indeed, in the original manuscript, Liddle et al. described the index case and her two siblings while Botero-Velez et al. studied the index case again at the age of 49 (20 months after kidney transplantation), in addition to 43 family members. They considered as affected 18 relatives presenting with arterial hypertension, as unaffected 15 normotensive subjects with an affected parent and ten not-at-risk subjects (partners and offspring of unaffected parents) [6]. A great variability in clinical features (hypertension severity, age at onset, plasma potassium concentration, urinary aldosterone excretion levels) suggested a variable penetrance of the disease [7]. Considering the clinical response to epithelial sodium channel inhibitor triamterene and the lack of improvement using mineralocorticoid receptor antagonist and a low sodium diet, it was hypothesized that the candidate gene could be involved in the pathway of sodium handling in the distal nephron. Thus, in 1994, a complete linkage of LS to the *SCNN1B* gene (encoding the β subunit of epithelial sodium channel, ENaC) was demonstrated and the first causative mutation was identified in Liddle’s original kindred as a premature stop codon, p.Arg566* (originally referred as p.Arg564* according to the homologous rat sequence) [7]. In the following years, several different germinal mutations in the *SCNN1A*, *SCNN1B* and *SCNN1G* genes, encoding, respectively, for the α, β and γ subunits of ENaC were identified, as described below.

### 2.2. Pathophysiology and Genetics

**Figure 1:**
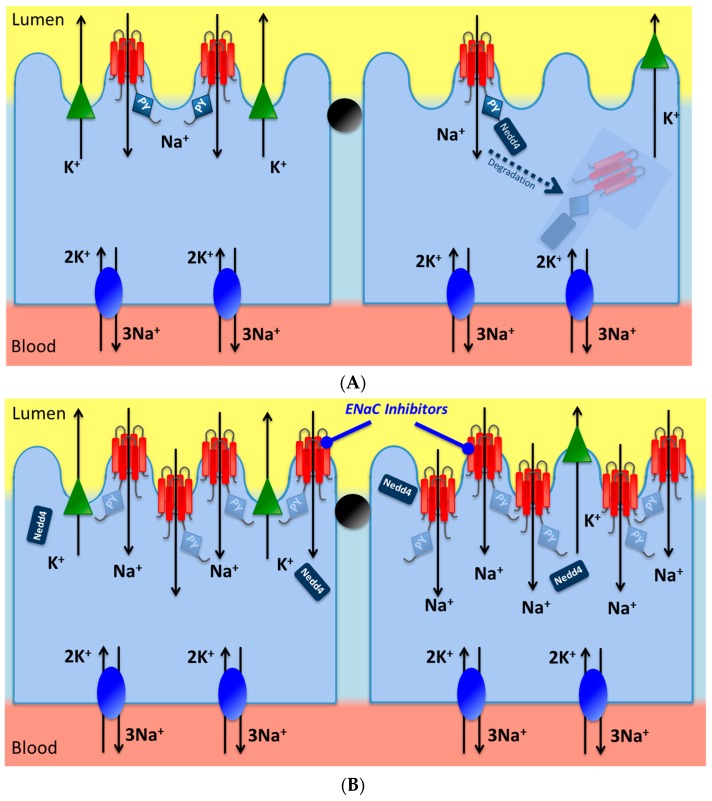

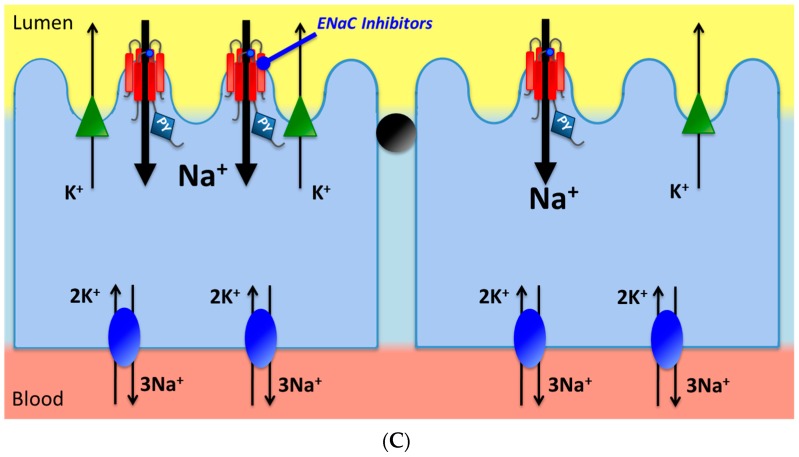
(**A**) Physiopathology of ENaC. Under physiological conditions, the epithelial Na^+^ channel (ENaC) is expressed on the luminal side of distal nephron epithelium. ENaC is positively regulated by aldosterone and antidiuretic hormone and allows the passage of Na^+^ ions from lumen toward cytoplasm. The proline-rich sequence (indicated as PY), located at the C-terminus of each subunit, regulates channel internalization and degradation, through Nedd4 binding and ubiquitination. ENaC function is combined with K^+^ channel ROMK (green triangles) and Na^+^/K^+^ ATPase (blue ovals) and it is crucial for hydroelectrolytic homeostasis, consisting in sodium renal reabsorption and potassium excretion; (**B**) β and γ subunits mutations. The germline mutations of the *SCNN1B* and *SCNN1G* genes causes the loss or disruption of proline-rich sequence that has a pivotal role in negative regulation of the channel. These mutations are gain-of-function and determine an increased membrane density of ENaC and a consequent increase in renal Na^+^ reabsorption; (**C**) α subunit mutation. The germline mutation of the *SCNN1A* gene affects the extracellular domain, causing the disruption of a disulphide bridge. It is a gain-of-function mutation that leads to an increase of the open probability of the channel and a consequent increase in Na^+^ current, without affecting the PY motif.

ENaC is a amiloride-sensitive epithelial sodium channel, localized in the apical portion of epithelial cells of distal nephron, distal colon, lung and ducts of exocrine glands [8]. Under physiological conditions, its expression and activity in the distal nephron are positively regulated by aldosterone and antidiuretic hormone and they are influenced by numerous extracellular factors, such as sodium, chloride, protons and proteases [9,10]. This channel is crucial, together with ROMK (renal outer medullary K^+^) channels and Na^+^/K^+^ ATPase, for Na^+^ reabsorption and, thus, for electrolytes homeostasis [9] (Figure 1A). The channel is a heteromeric complex constituted of three homologous subunits, α, β and γ [8,11,12], encoded by the *SCNN1A*, *SCNN1B* and *SCNN1G* genes, respectively. *SCNN1A* is located on chromosome 12p13.31, while *SCNN1B* and *SCNN1G* are located on chromosome 16p12.2 [9]. Although the α subunit alone is sufficient to induce a Na^+^ current, the expression of the three subunits induces a maximal amiloride-sensitive Na^+^ current [8]. The amino acid sequences of the three homologous subunits share 30–40% identity [8,9] and the protein structures are very similar, composed of two short intracellular N-terminus and C-terminus, two transmembrane domains (identified as TM1 and TM2) and a big extracellular loop [9,13]. Within the C-terminus of all three ENaC subunits, there is a highly conserved sequence, named the PY (Proline Tyrosine) motif [14]. This proline-rich sequence, PPxY, is a binding site for a member of the ubiquitin ligase family, Nedd4 (Neural precursor cell expressed, developmentally down-regulated 4), that mediates the internalization and the proteasomal degradation of the channel [9,14,15,16].

Liddle syndrome results from germline mutations in *SCNN1A*, *SCNN1B* or *SCNN1G* genes. The first mutation to be identified was the nonsense p.Arg566* substitution of the β subunit, in the large kindred described by Liddle et al. and subsequently by Botero-Velez et al. [4,6,7,17]. This mutation causes a truncation of the C-terminus of the β subunit with loss of the PY motif.

The first germinal mutation in the *SCNN1G* gene, resulting in the nonsense substitution p.Trp573*, was identified by Hansson et al. in 1995 [18]. Again, this mutation erases the γ subunit’s C-terminus, causing the loss of the PY motif. In the following years, 24 different mutations of the β subunit and six different mutations of the γ subunit were identified in 72 families from different countries (Table 1). The vast majority of the reported cases are determined by missense (ten different in 30 families), nonsense (eight in 21 families) or frameshift mutations (12 in 20 families) in *SCNN1B* or *SCNN1G* genes, that cause loss or disruption of the PY motif [9,19]. The loss of the proline-rich sequence prevents the internalization and degradation of the channel via the ubiquitination-proteasomal pathway and allows the accumulation of ENaC in the distal nephron apical membrane leading to an increase in sodium reabsorption [9,20,21]. The mutations are in fact responsible for an augmented apical membrane channel density and a subsequent increase in amiloride-sensitive inward sodium current, as demonstrated by in vitro studies in *Xenopus laevis* oocytes (Figure 1B) [21]. In 1996, Firsov et al. developed a quantitative method, based on the binding of a monoclonal antibody against a FLAG epitope inserted in the extracellular domain of α, β and γ subunits, and demonstrated a significant correlation between the entity of Na^+^ inward current and the number of ENaC on the cellular membrane [22].

Interestingly, additional mechanisms have been implicated in the augmented Na^+^ reabsorption, including an increase in channel open probability [23], an increase in the fraction of proteolitically cleaved channel (active) [24], together with a reduced feedback inhibition of ENaC by intracellular Na^+^ [25].

As an example, the reported mutation p.Asn530Ser in the γ subunit [44] which is located in the TM2 segment and does not affect the PY motif, causes a two-fold increase in amiloride-sensitive Na^+^ current, that was not associated to an increase in cell surface expression of the channel [44].

Recently, a germline mutation in the α subunit (p.Cys479Arg) was identified in a Caucasian family affected by Liddle syndrome (Table 1) [26]. This missense mutation is localized in the highly conserved extracellular domain of the subunit and leads to the disruption of a disulphide bridge. The p.Cys479Arg substitution increases the open conformation of the channel, resulting in a two-fold increase in Na^+^ current, without affecting channel density at the plasma membrane [26].

In vivo studies conducted on mice homozygous for the *SCNN1B* p.Arg566* mutation, indicate that the transition zone between the late distal convoluted tubule and the connecting tubule, is the main nephron site of ENaC hyperactivity in LS [69], where its activity is largely aldosterone independent [70]. However, ENaC is also expressed in several brain structures, including the supraoptic nucleus, magnocellular paraventricular nucleus, hippocampus, choroid plexus, ependyma, and brain blood vessels [71]. Mice lacking Nedd4-2 (Nedd4^−/−^) develop a phenotype of LS and display an increased ENaC expression in the central nervous system together with an increased blood pressure response after the infusion of Na^+^-rich cerebrospinal fluid compared to wild-type animals [72]. Similarly, Nedd4-2^−/−^ mice display a marked increase in cerebrospinal fluid Na^+^ concentration, following a high sodium diet. Both effects were largely prevented by the intra-cerebro-ventricular infusion of the ENaC blocker benzamil, raising the question as to whether a similar mechanism could be implicated in the pathogenesis of arterial hypertension in patients affected by LS as well [72].

Interestingly, specific β ENaC single nucleotide polymorphisms (SNPs) have been associated with arterial hypertension. In particular, the SNP rs3743966 in intron 12 (c.1543-112A>T) was significantly associated with essential hypertension in Chinese hypertensive families [73] and the intronic variants rs7205273 (c.-9+11091C>T) and rs8044970 (c.311+1599T>G) were associated with blood pressure in a large Korean population [74]. The missense SNPs (rs1799979, rs149868979 and rs1799980 leading to the substitutions p.Thr594Met, p.Arg563Gln and p.Gly442Val), have been found to be associated with arterial hypertension and with increased markers of Na^+^ channel activity [75,76,77,78]. In particular, the p.Thr594Met substitution was highly prevalent in a large population of black African origin, its frequency increased with the severity of hypertension [77] and was significantly associated with low plasma renin activity [76].The association of α ENaC polymorphisms (rs2228576, rs11542844, rs3741913) (resulting in the substitutions p.Thr663Ala, p.Ala334Thr and p.Cys618Phe) have been associated with high blood pressure in some studies, but not in others [79]. Functional studies in *Xenopus laevis* oocytes showed that the p.Cys618Phe and p.Ala663Thr polymorphisms (but not the p.Thr633Ala) increased channel activity by 3.3 and 1.6-fold, respectively [79]. Similarly, after different studies showed an association between *SCNN1G* locus and blood pressure variation [80,81], four *SCNN1G* intronic SNPs, rs13331086 (c.914-468T>G), rs11074553 (c.1077+2571G>A), rs4299163 (c.1077+3271C>G) and rs5740 (c.1176+14A>G) resulted to be associated to systolic blood pressure in the general Australian white population, after adjustment for age, sex and body mass index [82]. In particular, the association of rs13331086 was confirmed in a much larger cohort including more than 8000 individuals and the minor allele of this SNP was associated with a 1 mmHg increase in systolic blood pressure and 0.52 mmHg increase in diastolic blood pressure [83].

### 2.3. Diagnosis Prevalence and Phenotypes

The prevalence of Liddle syndrome across the general hypertensive population is unknown. In two recent studies, including 330 and 766 Chinese patients affected by arterial hypertension, after the exclusion of the most common secondary forms, the prevalence of Liddle syndrome resulted to be 1.52% (5/330) [32] and 0.91% (7/766) [27], respectively. Through genome-wide analysis, Pagani et al. demonstrated the presence of a common ancestor for three apparently unrelated Italian families carrying the p.Pro617Leu β mutation. Estimating the number of generations intervening between LS patients reported as unrelated, the authors suggested a much higher prevalence of LS than currently estimated [84].

The diagnosis of Liddle syndrome is based on *SCNN1A*, *SCNN1B* and *SCNN1G* gene sequencing. The genetic test is appropriate in the presence of early onset hypertension, hypokalemia, low renin and low aldosterone, with or without a positive family history. Genetic screening has to be performed also in first-degree relatives of a mutation carrier given the autosomal dominant inheritance (50% risk of transmission) and the variable phenotype reported in some families.

The typical clinical feature is resistant, early onset salt-sensitive arterial hypertension, often associated with a family history for early onset hypertension and sudden death. Biochemically, the characteristic findings are hypokalemia, metabolic alkalosis, suppressed PRA (plasma renin activity) and low serum aldosterone levels (Table 1). Hypertension results from increased Na^+^ reabsorption at the distal nephron level, leading to volume expansion, which is also responsible for the observed biochemical phenotype of low renin and low serum aldosterone. At the cellular level, following ENaC opening, 3 Na^+^ ions are actively exchanged for 2 K^+^ ions across the basolateral membrane by the Na^+^/K^+^-ATPase (Figure 1A), which exit the apical membrane through different K^+^ channels and are lost in the urine (resulting in hypokalemia and metabolic alkalosis) [85]. Other signs and symptoms frequently reported arise as a consequence of hypokalemia and include muscular weakness, polyuria (as low K^+^ concentrations in the tubular fluid prevent the Na^+^/2Cl^−^/K^+^ pump of the thick ascending limb of the loop of Henle and the Na^+^/K^+^ pump of the collecting duct from working properly [86] and downregulate aquaporin-2 channels [87]), polydipsia (secondary to polyuria), and as a consequence of hypertension, including headache, dizziness, retinopathy, chronic kidney disease, left ventricular hypertrophy and sudden death (supposed to be caused by malignant arrhythmias elicited by severe hypokalemia).

However, extremely severe phenotypes and mild forms can coexist, with some patients carrying a causative mutation who are normotensive (Table 1) or in whom a clinical diagnosis of LS was made in old age [88]. Systematic review of the reported cases revealed that hypertension is present in 92.4% of the patients, hypokalemia (defined as serum K^+^ <3.5 mmol/L) in 71.8% and hypoaldosteronemia (defined as serum aldosterone <5 ng/dL) in 58.2% of the cases. As reported for other forms of monogenic hypertension [89,90], this variability is not only observed between kindreds carrying different mutations, but also between affected members of the same family (Table 1). It is likely that both environmental and genetic factors, including Na^+^ intake and polymorphisms in genes involved in Na^+^ handling could influence the phenotypic manifestation of the disease [63].

The variable expression of the clinical phenotype can hamper the diagnosis of Liddle syndrome, that might be overlooked in patients with a mild clinical manifestation.

The specific treatment of LS is represented by K^+^-sparing diuretics amiloride and triamterene, that are ENaC blockers. According to the pathophysiology, the efficacy of the ENaC blockers is enhanced by dietary low salt intake (2 g NaCl/day) [54]; indeed, the competition between these molecules and sodium at the level of the ENaC ionic pore is well known [17]. ENaC blockers are effective in normalizing both blood pressure and the typical biochemical alterations (hypokalemia, suppressed PRA and low aldosterone level). Monitoring serum electrolytes during therapy is worthwhile, although the incidence of hyperkalemia is rare if renal function is normal and potassium intake is not excessive [17]. In most countries these drugs are commercialized only in association with thiazide or loop diuretic and the fixed doses could be a disadvantage in titrating therapy. Amiloride appears to be a safe and effective medication in pregnancy in reaching optimal blood pressure values and normal kalemia [42,50]. Neither hypertension nor hypokalemia improve under treatment with spironolactone (since activation of the mineralocorticoid receptor is not implicated in Na^+^ reabsorption) and this might represent an additional clinical criterion to suspect Liddle syndrome [91].

## 3. Description of a New Case of Liddle Syndrome

The index case is a 13-year-old Caucasian boy referred to our Hypertension Unit by the Pediatric Endocrinology Department for arterial hypertension that had been diagnosed six months before. His mother was normotensive and in general good health. His father, affected by arterial hypertension and hypokalemia, died at the age of 38 of sudden cardiac death. The patient was born at term from an uneventful pregnancy to non-consanguineous parents and his past medical history was unremarkable, with no clinical signs of abnormal sexual development. On physical examination, his height and weight were 165 cm (86th percentile for age and sex) and 55 kg (80th percentile for age and sex), respectively. At diagnosis, blood pressure was 184/109 mmHg (>99th percentile for age, gender and height (SBP, 90th percentile: 125 mmHg; 95th percentile: 129 mmHg; 99th percentile: 136 mmHg. DBP, 90th percentile: 79 mmHG; 95th percentile: 83 mmHg; 99th percentile: 91 mmHg) [92] and serum potassium was 3.2 mmol/L (normal range 3.5–5.0 mmol/L). Assessment of target organ damage revealed neither left ventricular hypertrophy nor microalbuminuria.

The patient was investigated to exclude secondary forms of hypertension. Low PRA (<0.1 ng/mL/h) and low serum aldosterone were detected on different occasions (1.0–5.9 ng/dL). 17-OH-progesterone, dehydroepiandrosterone, 4-δ-androstenedione, urinary cortisol and urinary androgen catabolites resulted in normal range for sex and age. The urinary steroid profile for apparent mineralocorticoid excess syndrome resulted negative (tetra-hydrocortisol + allo-tetra-hydrocortisol/tetra-hydrocortisone = 1.45). After three months of therapy with spironolactone (50 mg daily) without clinical and biochemical response, Liddle syndrome was hypothesized. The patient was treated with amiloride (5 mg daily) that successfully controlled blood pressure (120/65 mmHg) and normalized plasma K^+^ (4.8 mmol/L). The diagnosis of Liddle syndrome was confirmed by genetic analysis that identified the β ENaC germline mutation p.Pro618Leu. Considering the clinical presentation of the index case’s father, the inheritance of the mutation by paternal lineage is highly probable. However, a DNA sample from his father was not available.

## 4. Conclusions

Liddle syndrome is genetic autosomal dominant form of low renin arterial hypertension caused by germline mutations in the *SCNN1A*, *SCNN1B* and *SCNN1G* genes, encoding, respectively, the α, β and γ subunits of the epithelial sodium channel ENaC. Despite the typical phenotype presenting with severe hypertension and hypokalemia, the disease can be clinically heterogeneous, even with mild phenotypes. Herein, we report a new case caused by the germline p.Pro618Leu mutation of the gene *SCNN1B*. The index case presented with high blood pressure and hypokalemia at the age of 13 and a family history of sudden death. Hypertension and hypokalemia were well controlled by amiloride.

Considering the frequency of early-onset hypertension and severity of correlated complications, a well-timed diagnosis of LS is very important in order to administer the proper therapy.

In conclusion, further studies are needed to better define the clinical manifestations and the real prevalence of LS, an example of actionable genetic disease that warrant a proper therapy in order to prevent target organ damage and associated cardiovascular complications.

## Figures and Tables

**Table 1:** Clinical and biochemical phenotype of patients affected by Liddle’s syndrome. *—in the original manuscript the mutation is reported according to the homologous rat sequence, HT—hypertension, SD—sudden death, LVH—left ventricular hypertrophy, TOD—target organ damage, CV—cardio vascular, n.a.—not available. Hypokalemia defined as serum K^+^ <3.5 mmol/L, hypoaldosteronemia defined as serum aldosterone <5 ng/dL or urinary aldosterone <5 µg/24 h.

Study	Country	Families (*n*)	Patients (Genetic/Clinical Diagnosis)	Sex (M/F, not Available)	Hypertension (n/tot Available)	Spontaneous Hypokalemia (n/tot Available)	Low Aldosterone (n/tot Available)	Reported Symptoms/TOD/CV Events/Other
*SCNN1A* mutations (NM_001038.5→NP_001029.1 isoform 1)								
p.Cys479Arg								
Salih M. (2017) [26]	The Netherlads	1	2/0	1/1	1/2	1/1	2/2	None
*SCNN1B* mutations (NM_000336.2→NP_000327.2)								
p.Gln564*								
Liu K. (2017) [27]	China	1	1/0	1/0	1/1	0/1	0/1	Stroke
p.Arg566*								
Shimkets R.A. (1994) [7] *	USA	2	19/4	10/8, 1	19/19	3/3	3/3	Renal failure, history of juvenile CV accidents
Melander O. (1998) [28] *	Sweden	1	6/0	2/4	4/6	2/6	3/5	None
Kyuma M. (2001) [29] *	Japan	1	3/0	2/1	3/3	2/3	0/1	Muscular weakness, retinopathy
Shi J.Y. (2010) [30] *	China	1	1/0	1/0	1/1	1/1	1/1	History of stroke
Gong L. (2014) [31]	China	1	3/0	2/1	3/3	3/3	0/1	LVH
Wang L.P. (2015) [32]	China	1	1/0	1/0	1/1	1/1	0/1	None
Polfus L.M. (2016) [33]	USA	1	2/0	1/1	2/2	1/1	1/1	Asthenia, palpitation, LVH, proteinuria
Cui Y. (2017) [34] *	China	3	3/6	½	3/3	3/3	0/3	Dizziness, headache, history of SD and stroke
Liu K. (2017) [27]	China	1	3/0	2/1	3/3	3/3	0/3	LVH
p.Gln568*								
Cui Y. (2017) [34]	China	1	1/0	1/0	1/1	1/1	0/1	Dizziness, headache
p. Ser570Tyrfs*589								
Freerks R. (2017) [35]	South Africa (Black origin)	1	1/0	1/0	1/1	1/1	1/1	Headache, muscle fatigue, exertional dyspnoea, retinopathy, LVH
p.Pro575Argfs*591								
Phoojaroenchanachai M. (2015) [36]	Thailand	1	2/1	1/1	2/2	2/2	1/1	Lightheadedness, proximal muscle weakness
p. Ala579Leufs*582								
Jeunemaitre X. (1997) [37]	France	1	4/0	3/1	4/4	4/4	4/4	History of SD
p.Gln591*								
Shimkets R.A. (1994) [7] *	USA	1	1/0	0/0, 1	1/1	0/0	0/0	n.a.
p.Thr594Hisfs*607								
Shimkets R.A. (1994) [7] *	USA	1	1/0	0/0, 1	1/1	0/0	0/0	n.a.
p.Ala595Argfs*607								
Findling J.W. (1997) [38] *	USA	1	8/2	1/7	5/8	2/7	7/7	Myocardial infarction
p.Arg597Profs*607								
Inoue T. (1998) [39]	Japan	1	2/4	0/2	2/2	1/2	2/2	None
Jackson S.N. (1998) [40] *	UK	1	4/1	3/1	3/4	1/2	4/4	None
Nakano Y. (2002) [41] *	Japan	1	1/0	0/1	1/1	1/1	1/1	None
Gong L. (2014) [31]	China	1	1/1	0/1	1/1	1/1	0/1	Dizziness, chronic kidney disease, SD, history of stroke
Awadalla M. (2017) [42]	USA (Black origin)	1	1/6	0/1	1/1	1/1	1/1	Proteinuria
p.Arg597Alafs*675								
Shimkets R.A. (1994) [7] *	USA	1	2/0	0/2	2/2	0/0	0/0	n.a.
p.Thr601Aspfs*607								
Ma X. (2001) [43]	China	1	8/0	5/3	8/8	4/8	8/8	Fatigue, headache, nycturia, history of cerebral hemorrhage
Hiltunen T.P. (2002) [44]	Finland	1	4/0	1/3	3/4	1/1	0/0	None
p.Pro603Alafs*607								
Cui Y. (2017) [34]	China	2	3/0	3/0	3/3	3/3	0/3	Headache, dizziness, history of stroke and SD
p.Pro616Leu								
Gao L. (2013) [45] *	China	1	7/3	5/2	7/7	7/7	0/7	Tachycardia, LVH, history of stroke
Liu K. (2017) [27]	China	3	7/0	2/5	7/7	5/7	1/7	LVH
Kuang Z.M. (2017) [46]	China	1	2/1	1/1	2/2	1/2	0/1	Muscular weakness, history of cerebral hemorrhage
p.Pro617His								
Sawathiparnich P. (2009) [47] *	Thailand	1	4/2	0/4	4/4	1/2	3/3	Headache, LVH
p.Pro617Leu								
Rossi E. (2008) [48]	Italy	1	1/2	1/0	1/1	1/1	1/1	LVH
Rossi E. (2011) [49]	Italy	1	4/4	2/2	4/4	0/4	4/4	LVH
Caretto A. (2014) [50]	Italy	1	4/1	1/3	3/3	1/3	2/2	Headache, visual scotoma, fetal growth retardation, history of stroke and cerebral hemorrhage
p.Pro617Ser								
Inoue J. (1998) [51] *	Japan	1	4/2	4/0	1/4	3/4	0/0	None
Cui Y. (2017) [34]	China	1	1/0	0/1	1/1	1/1	0/1	Headache, dizziness
p.Pro617Serfs*621								
Cui Y. (2017) [34]	China	1	1/0	1/0	1/1	0/1	0/1	Headache, dizziness
p.Pro618His								
Freundlich M. (2005) [52]	USA (Black)	1	2/0	1/1	2/2	0/1	1/1	None
Wang W. (2006) [53] *	China	1	5/0	2/3	4/5	5/5	1/1	Muscular weakness
Yang K.Q. (2018) [54]	China	1	6/2	3/3	4/6	4/6	6/6	Syncope, microalbuminuria, LVH, headache, history of stroke
p.Pro618Leu								
Hansson J.H. (1995) [55] *	USA (Black origin)	1	3/0	1/2	3/3	3/3	2/2	Stroke
Uehara Y. (1998) [56] *	Japan	1	1/0	0/1	1/1	1/1	1/1	None
Takeda I. (1999) [57] *	Japan	1	1/0	0/0, 1	1/1	1/1	0/0	n.a.
Gao P.J.(2001) [58] *	China	1	5/2	4/1	4/5	1/5	1/5	History of stroke and SD
Yamashita Y. (2001) [59] *	Japan	1	1/0	1/0	1/1	0/0	0/0	n.a.
Cui Y. (2017) [34]	China	2	2/0	2/0	2/2	2/2	0/2	None
Büyükkaragöz B. (2016) [60]	Turkey	1	5/0	1/4	5/5	3/5	4/4	Headache, dizziness, LVH, retinopathy, history of SD
This manuscript	Italy	1	1/1	1/0	1/1	1/1	0/1	History of SD
p.Pro618Arg								
Ciechanowicz A. (2005) [61] *	Czech Republic	1	2/0	2/0	2/2	2/2	2/2	None
Furuhashi M. (2005) [62] *	Japan	1	2/0	0/2	1/2	2/2	2/2	Asthenia
p.Pro618Ser								
Uehara Y. (1998) [56] *	Japan	1	2/2	1/1	2/2	2/2	1/2	None
Bogdanović R. (2012) [63]	Serbia	1	3/3	2/1	3/3	1/3	3/3	LVH, history of SD
Wang L.P. (2012) [64] *	China	1	3/0	0/3	3/3	3/3	0/3	None
p. Asn619Glnfs*621								
Yang K.Q. (2015) [65]	China	1	1/0	1/0	1/1	1/1	1/1	None
Cui Y. (2017) [34]	China	1	1/0	1/0	1/1	1/1	0/1	Headache, dizziness, history of stroke
Liu K. (2017) [27]	China	1	1/0	1/0	0/1	1/1	1/1	Mild impairment of renal function
p.Tyr620His								
Tamura H. (1996) [66] *	Japan	1	5/0	3/2	5/5	2/4	4/4	Chronic kidney disease
*SCNN1G* mutations (NM_001039.3→NP_001030.2)								
p.Asn530Ser								
Hiltunen T.P. (2002) [44]	Finland	1	2/0	1/1	2/2	1/2	0/1	None
p.Gln567*								
Shi J.Y. (2010) [30]	China	1	3/0	2/1	1/1	1/1	0/1	n.a.
Zhang P. (2017) [67]	China	1	1/0	1/0	1/1	1/1	1/1	Muscular weakness, polyuria, polydipsia, LVH
p.Glu571*								
Wang L.P. (2015) [32]	China	1	6/1	2/4	5/6	6/6	0/6	History of stroke
Liu K. (2017) [27]	China	1	5/0	1/4	5/5	5/5	0/5	Stroke, aortic stenosis
p.Trp573*								
Hansson J.H. (1995) [18] *	Japan	1	6/0	2/4	6/6	5/6	4/6	Leg numbness
p.Trp575*								
Yamashita Y. (2001) [59] *	Japan	1	1/0	0/0, 1	1/1	0/0	0/0	n.a.
p.Glu583Aspfs*585								
Wang Y. (2007) [68]	China	1	1/0	1/0	1/1	1/1	0/1	None
TOTAL								
	-	72	200/51	97/98, 5	182/197 (92,4%)	117/163 (71,8%)	85/146 (58,2%)	-
